# Usefulness of chest ultrasonography in the management of acute respiratory failure in the emergency room

**DOI:** 10.1186/cc9448

**Published:** 2011-03-11

**Authors:** S Silva, M Dao, C Biendel, B Riu, J Ruiz, B Bataille, J Bedel, M Genestal, O Fourcade

**Affiliations:** 1 CHU Toulouse Purpan, Toulouse, France

## Introduction

Acute respiratory failure does not always present in conditions that are ideal for immediate diagnosis, which sometimes compromises outcome. Physical examination and bedside radiography are imperfect, resulting in a need for sophisticated test results that delay management. Recently, a decision tree utilizing bedside ultrasonography has been proposed to guide diagnosis of severe dyspnoea. This study examines the relevance of this approach to diagnose acute respiratory failure in the emergency room (ER).

## Methods

This prospective study was conducted in university teaching hospitals over 1 year investigating 59 consecutive adults patients admitted to the ER with acute respiratory failure. At arrival, two diagnosis approaches have been performed: Standard (established using standardized tests and not including ultrasound data), and Ultrasound (derived from the ultrasound decision tree). Investigators did not participate in patient management, and were blinded to the data from the other group. We compared diagnosis results from both approaches (Standard and Ultrasound) with the official diagnosis established at the end of the hospitalization by the ER staff. The internal review board of the hospital approved this study. The MacNemar test was used to analyse the error rate. The means were compared using Student's *t *test.

## Results

The error rates were 30% and 10% in the Standard and Ultra-sound groups, respectively (MacNemar test, *P *< 0.02). The number of erroneous initial diagnoses was significantly greater using conventional tools in patients with pneumonia and pulmonary oedema (Standard vs. Ultrasound, *P *< 0.05). More patients received inappropriate therapy in the Standard than in the Ultrasound group (35% vs. 15%, *P *< 0.05).

## Conclusions

Ultrasound generates standardized and reproducible patterns, which have been proposed to help bedside diagnosis in patients admitted to the ER with acute respiratory failure. Our data highlight a significant improvement of initial diagnosis accuracy using this tool. Chest ultrasound performed by physicians in charge of ERs appears to be one of the most promising techniques for management of patients admitted to the ER with acute respiratory failure and should rapidly expand in the near future.
